# Historical Invasion Records Can Be Misleading: Genetic Evidence for Multiple Introductions of Invasive Raccoons (*Procyon lotor*) in Germany

**DOI:** 10.1371/journal.pone.0125441

**Published:** 2015-05-06

**Authors:** Mari L. Fischer, Axel Hochkirch, Mike Heddergott, Christoph Schulze, Helena E. Anheyer-Behmenburg, Johannes Lang, Frank-Uwe Michler, Ulf Hohmann, Hermann Ansorge, Lothar Hoffmann, Roland Klein, Alain C. Frantz

**Affiliations:** 1 Department of Biogeography, Trier University, Trier, Germany; 2 Musée National d'Histoire Naturelle, Luxembourg, Luxembourg; 3 Landeslabor Berlin-Brandenburg, Frankfurt (Oder), Germany; 4 Lower Saxony State Office for Consumer Protection and Food Safety, Food and Veterinary Institute Braunschweig/Hannover, Hannover, Germany; 5 Institut für Tierökologie und Naturbildung, Gonterskirchen, Germany; 6 Dresden University of Technology, Institute of Forest Botany and Forest Zoology, Tharandt, Germany; 7 Department of Wildlife Ecology, Research Institute for Forest Ecology and Forestry Rhineland-Palatinate, Trippstadt, Germany; 8 Senckenberg Museum of Natural History Görlitz, Görlitz, Germany; 9 Thüringer Landesamt für Verbraucherschutz, Bad Langensalza, Germany; Chinese Academy of Sciences, CHINA

## Abstract

Biological invasions provide excellent study systems to understand evolutionary, genetic and ecological processes during range expansions. There is strong evidence for positive effects of high propagule pressure and the associated higher genetic diversity on invasion success, but some species have become invasive despite small founder numbers. The raccoon (*Procyon lotor*) is often considered as a typical example for such a successful invasion resulting from a small number of founders. The species’ largest non-native population in Germany is commonly assumed to stem from a small number of founders and two separate founding events in the 1930s and 1940s. In the present study we analyzed 407 raccoons at 20 microsatellite loci sampled from the invasive range in Western Europe to test if these assumptions are correct. Contrary to the expectations, different genetic clustering methods detected evidence for at least four independent introduction events that gave rise to genetically differentiated subpopulations. Further smaller clusters were either artifacts or resulted from founder events at the range margin and recent release of captive individuals. We also found genetic evidence for on-going introductions of individuals. Furthermore a novel randomization process was used to determine the potential range of founder population size that would suffice to capture all the alleles present in a cluster. Our results falsify the assumption that this species has become widespread and abundant despite being genetically depauperate and show that historical records of species introductions may be misleading.

## Introduction

Alien invaders are of evolutionary interest because of the role of genetic processes during their establishment and spread. While introduced populations are normally [1]—but not always [2]—genetically impoverished compared to the source population, high levels of genetic variation are assumed to increase an introduced species’ potential for adaptation during initial establishment and range expansion [3]. However, reduced genetic diversity *per se* does not prevent invasion as there are many examples of genetically depauperate invaders [1, 4]. Indeed, it has been shown that the importance of genetic diversity for invasion success can be reduced, if species are pre-adapted to become invasive elsewhere [5] or if phenotypic plasticity rather than adaptation contributes to successful colonisation [6]. Generally, there seems to be a positive correlation between propagule pressure and invasion success [7], which has also been confirmed in terrestrial vertebrate species [8, 9]. Besides reducing the impact of stochastic events, high propagule pressure and multiple sources of introduction can help to overcome genetic founder events in introduced populations [1, 3].

The raccoon (*Procyon lotor*) is a medium-sized Central and North American carnivore that has colonized different parts of the world due to deliberate or accidental releases [10–12]. The species is particularly abundant and widespread in Germany, where it was first introduced to Europe in the 1930s [13]. It is commonly assumed that the whole German population derives from two separate founding events in the 1930s and 1940s [14–16]. In 1934, two raccoons of each sex were released near lake Edersee (Hesse, central Germany), probably supplemented by additional escapees in the 1940s [13, 17]. A second population became established in north-eastern Germany after 25 individuals escaped from a fur farm in Wolfshagen (Brandenburg) in 1945 [18]. According to hunting statistics, the German raccoon population has dramatically increased in abundance over the last twenty years, from just over 3,000 harvested individuals in 1995 to around 100,000 in 2014 [19]. While there is only limited evidence of a negative ecological impact of the presence of raccoons in Europe [20], the recent rise in densities is likely to increase the risk of pathogen transmission to humans, wildlife and domestic animals [21].

As raccoons are omnivorous generalists that can live in forested areas as well as in urban habitats [16], genetic diversity might not be a short-term pre-requisite for raccoons to become successful invaders. If the population derived from a small number of individuals, it must have undergone a genetic bottleneck during its foundation. Indeed, Frantz et al. [22] only found five different haplotypes of the mitochondrial control region in 193 raccoons sampled from Germany (and neighbouring countries), while 76 different haplotypes were observed in 311 samples from the eastern United States [23]. Similar results are reported from central Spain, where two introduction events with two to four founders per population have been documented [24].

However, accidental or deliberate releases of raccoons kept as household pets are fairly common [11, 12] and it is possible that the number of founders of the German raccoon population (and hence propagule pressure) was greater than commonly assumed. Single reports [18] and recent genetic studies already suggested that the invasion history of German raccoons might be more complex than often assumed. For example, mitochondrial DNA (mtDNA) sequencing provided evidence for presence of a third founder population in eastern Germany [22]. Furthermore, a recent microsatellite-based parentage study [25] suggested a relatively high genetic diversity—at least compared to many other invasive mammals [1]—of raccoons in north-eastern Germany (6.2 alleles/locus, observed heterozygosity *H*_*O*_ = 0.62). However, North American-based studies frequently report more than ten alleles and values of *H*_*O*_>0.70 for many loci [26–28].

We genotyped 20 microsatellite loci to analyse the genetic diversity and population structure of German raccoons. Our overall objective was to test whether raccoons became established and reached high densities in Germany despite being genetically impoverished, or whether a larger pool of founders contributed to a genetically diverse population. Specifically, we wanted to reconstruct the number and geographic locations of founder populations and assess the degree of admixture between them. We used genetic assignment and exclusion methods to test for the presence of recently escaped or released individuals. Furthermore, we inferred the number of founder individuals in each population by estimating the number of individuals required to introduce all the alleles identified.

## Materials and Methods

### Laboratory work

**Fig 1:**
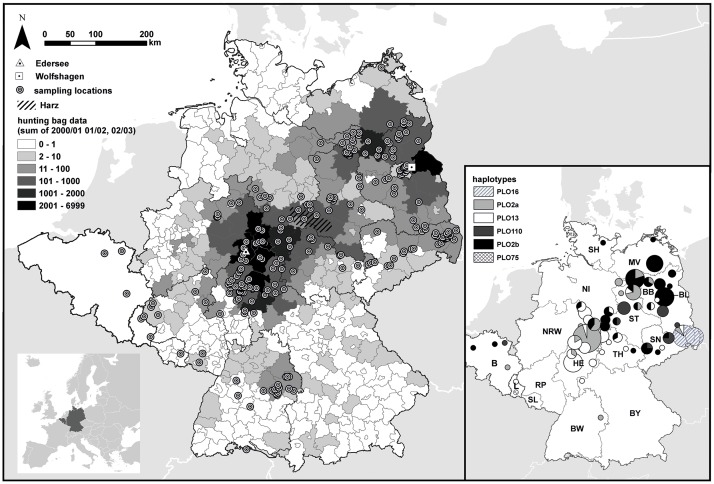
Geographic origin of the 407 raccoon samples used in the present study. One point can represent multiple samples. Hunting bag data: the number of individuals harvested between 2000/01 and 2002/03 in German administrative districts (light grey lines). Inset: mtDNA haplotype frequency distribution of a subset of 193 of the 407 raccoons [19]. Size of the pies corresponds to the number of samples in the administrative districts. Samples were collected in 14 of the 16 federal states in Germany: Brandenburg (BB), Berlin (BL), Baden-Württemberg (BW), Bavaria (BY), Hesse (HE), Mecklenburg-West Pomerania (MV), Lower Saxony (NI), North Rhine-Westphalia (NRW), Rhineland-Palatinate (RP), Schleswig-Holstein (SH), Saarland (SL), Saxony (SN), Saxony-Anhalt (ST) and Thuringia (TH). The inset also shows the location of Belgium (B) and Luxembourg (L).

A total of 407 raccoon samples (tissue n = 336, hair n = 56, buccal swabs n = 15) was collected from the core distribution in Germany (near the introduction sites of Edersee and Wolfshagen) as well as from the expanding range margins in Germany, Belgium and Luxembourg (Fig 1). Samples were collected from trapped, road-killed or legally hunted individuals. As invasive species, raccoons are legally considered to be a game species in Germany (German federal hunting law: Bundesjagdgesetz §2 Abs.1) and can therefore be harvested by licensed hunters outside the closed season without special permission. No animal was killed with the aim of providing samples for this study. All hunted individuals were legally shot and made available to the authors. All non-German samples originated from road-killed individuals. No special permit was required to take samples from road-killed individuals.

Buccal swabbing was performed with sterile diagnostic rayon dry swabs (Copan Diagnostics Inc., Murrieta, CA, USA). Swabs were stored in sterile tubes at -20°C. Tissue samples were stored frozen or in absolute ethanol. Hair samples were stored in filter paper with silica gel (ThoMar OHG, Lütau, Germany) at room temperature. Genomic DNA from tissue or swab samples was extracted using the DNeasy Blood and Tissue kit (QIAGEN Hilden, Germany) following the manufacturer’s protocol (replacing ATL buffer with 200 μl PBS buffer for the swab samples). We extracted the hair DNA with a modified Chelex100 protocol, using 250 μl of a 10% Chelex100 Resin solution (BioRad, München, Germany) with addition of 4 μl Proteinase K (18 mg/ml) per sample and an overnight lysis step [29].

All individuals were genotyped at 21 microsatellite loci: *PLOT-01*, *PLOT-02*, *PLOT-03*, *PLOT-04*, *PLOT-05*, *PLOT-06*, *PLOT-07*, *PLOT-08*, *PLOT-10*, *PLOT-11*, *PLOT-13* [30], *PLO-M15*, *PLO2-117*, *PLO-M3*, *PLO-M20*, *PLO2-14*, *PLO-M2*, *PLO-M17*, *PLO3–86* [31], *PLM01* and *PLM03* [32]. All loci were co-amplified in seven multiplex polymerase chain reactions (PCR). Multiplex 1 contained loci *PLOT-01*, *PLOT-02*, *PLOT-03*, *PLOT-04*, multiplex 2 loci *PLOT-05*, *PLOT-06*, *PLOT-07*, *PLOT-08* and multiplex 3 loci *PLOT-10*, *PLOT-11*, *PLOT-13*. Multiplex 4 contained loci *PLO-M15*, *PLO2-117*, multiplex 5 loci *PLO-M3*, *PLO-M20*, *PLO2-14*, *PLO-M2* and multiplex 6 loci *PLO-M17*, *PLO3–86*. Multiplex 7 contained loci *PLM01*, *PLM03*. Reactions were performed in a final volume of 10 μl containing 1–10 ng DNA, 5 μl Type-it Microsatellite PCR master mix (QIAGEN Hilden, Germany) and 0.3 μM of each primer. PCRs were performed in a Multigene Gradient Thermal Cycler (Labnet International Inc., Edison, NJ, USA) using the following program: one cycle of 15 min at 95°C, 32 cycles at 94°C for 30s, 90s at the multiplex-specific annealing temperature (60.5°C for Multiplex 1, 63°C for Multiplexes 2 and 3, 58.5°C for Multiplexes 4 and 7, 55.5°C for Multiplexes 5 and 6), followed by 40s at 72°C and a final extension for 30 min at 72°C. Hair and swab samples were amplified and scored twice to minimalize the risk of genotyping errors. The fluorescently labelled PCR products were analysed on a MEGABACE 1000 automated sequencer (GE Healthcare, Freiburg, Germany) and fragment lengths of the alleles were scored by eye using the Fragment Profiler 1.2 (Amersham Bioscience, Freiburg, Germany). Preliminary data analysis (results not shown) provided evidence for linkage disequilibrium between *PLOT-04* and *PLO-M17*. We therefore excluded *PLO-M17* from all analyses.

### Genetic structure and descriptive statistics

We analysed the population genetic structure using two Bayesian genetic clustering algorithms. First, we analysed the data in STRUCTURE 2.3.4 [33]. We estimated the number of genetic subpopulations (*K*) by performing ten independent runs of *K* = 1–12 with 10^6^ Markov chain Monte Carlo (MCMC) iterations after a burn-in period of 10^5^ iterations, using the model with correlated allele frequencies and assuming admixture. ALPHA, the Dirichlet parameter for the degree of admixture, was allowed to vary between runs. After deciding on the most probable number of sub-populations based on the log-likelihood values (and their convergence) associated with each *K*, as well as on the Δ*K* method by Evanno et al. [34], we calculated each individual’s percentage of membership (*q*), averaging *q* over different runs of the same *K*. In order to facilitate geographical representation, the average *q* values for each administrative district (‘Landkreis’) were calculated and mapped using ArcGIS 10.1 (ESRI Inc., Redlands, CA, USA). Second, we also analysed our data using the ‘clustering of individuals’ algorithm implemented in BAPS v.6.0 [35], which infers the number of genetic clusters in a data set. We performed ten runs for each of *K* = 2–12.

For the subsequent analyses, populations were pre-defined by placing samples into the STRUCTURE cluster for which they showed the highest percentage of membership (*q*). We represented the results from *K* = 7, averaging *q* over eight runs with the highest log-likelihood values (see Results). We tested for the significance of heterozygote deficiency or excess [36] with the Markov chain method in Genepop 4.1.4 [37], with 10,000 dememorization steps, 500 batches and 10,000 subsequent iterations. Pairs of loci were tested for linkage disequilibrium using an exact test based on a Markov chain method as implemented in Genepop 4.1.4 The false discovery rate technique was used to eliminate false assignment of significance by chance [38].

We visualised the genetic differentiation among the samples with a Factorial Correspondence Analysis (FCA) in Genetix 4.05.2 [39] and performed genetic exclusion tests in the program GENECLASS 2.0 [40] to test the hypothesis that individuals assigned to a specific cluster but visualized as outliers in the FCA were in fact individuals that had recently been introduced to the population. Exclusion probabilities were calculated with the Monte Carlo method of Paetkau et al. [41] by simulating 10,000 multi-locus genotypes and by setting the threshold for exclusion of individuals to 0.001 [42]. The level of genetic differentiation between the genetic clusters was quantified with *F*_ST_ [35] in GenAlEx version 6.501 [43] and by an Analysis of molecular variance (AMOVA) using 9,999 permutations.

We tested the data set for isolation-by-distance (IBD) by analysing genetic relatedness between pairs of individuals as a function of geographical distance, using program SPAGeDi 1.2 [44]. The slope of this relationship offers a convenient measure of the degree of spatial genetic structuring. As suggested by Vekemans & Hardy [45], the Loiselle kinship coefficient (*F*_ij_) [46] was chosen as a pairwise estimator of genetic relatedness, as it is a relatively unbiased estimator with low sampling variance. The slope was tested for a significant difference from zero by 10 000 permutations of locations of individuals. We performed an analysis on the whole data set, as well as on pairs of individuals assigned to the same STRUCTURE cluster only (using cluster-specific allele frequencies).

We used GenAlEx to estimate the number of alleles (*A*), observed heterozygosity (*H*_O_) and unbiased expected heterozygosity (_u_ *H*_E_) for each STRUCTURE cluster and the number of private alleles (_p_ *A*) in a cluster. Allelic richness (*A*_R_) was calculated using Fstat 2.9.3.2 [47]. Estimates were based on a minimum sample size of 13 diploid individuals. Relatedness coefficients were calculated in COANCESTRY 1.0.1.2. [48], which provides the Triadic Maximum Likelihood estimator TrioML based on Wang [49], estimating pairwise relatedness (*r*) by the use of a third individual as a reference, thus reducing the chance of genes identical in state being mistakenly inferred as identical by descent. We estimated effective population sizes (*N*_e_) of each genetic cluster using the linkage disequilibrium method in program NeEstimator v.2.01 [50], estimated 95% confidence intervals using jackknifing and excluded rare alleles with frequencies less than 0.02.

### Calculating minimum number of founders

There are several methods available to estimate the number of founders based on genetic data [51–54]. However, these methods require genetic information from the source population and assume no admixture between introduced populations. Our data set does not fulfil either condition. We therefore expanded an approach by Rasner et al. [55] and attempted to estimate the minimum number of founders required to introduce all empirically observed microsatellite alleles into each inferred STRUCTURE cluster. The genetic profiles of the founder individuals were generated by resampling the alleles in the empirical data set or by simulating genetic profiles based on allele frequencies. Custom-written scripts (S1 Script, S2 Script, S3 Script) for program R 3.1.0 [56] simulated different numbers of founder genotypes and ran 1,000 replicates for each founder size in order to estimate the smallest number of individuals whose genotypes contain all the alleles identified at the 20 microsatellite loci (assuming no physical linkage between loci and no past mutations). We considered the smallest minimum number of founders to be the simulated sample size that had a probability ≥ 0.05 of capturing all the observed alleles [55]. In order to account for the impact of sampling bias and the effect of genetic drift in small founder populations, we varied the R scripts in terms of the mechanisms for simulating founder genotypes: (1) we simulated genetic profiles by randomly sampling alleles—independently for each locus—without replacement from the genetic profiles present in the data set. (2) We simulated genetic profiles by resampling from the allele frequency distribution at each locus. (3) We simulated genetic profiles by resampling from allele frequency distributions assuming equal frequencies at each locus. While based on an oversimplified approach, this latter analysis was performed in an attempt to account for genetic drift. The maximum number of simulated individuals corresponded to the maximum amount of the individuals in a particular STRUCTURE cluster, except in the case of analysis 2, where threefold the maximum amount of individuals was simulated. In the case of the first two analytical approaches, we repeated the analysis after removing all genetic profiles that contained alleles that occurred at low frequency (<0.02) in the respective cluster [57] to avoid recently immigrated or admixed individuals inflating the estimate of the minimum number of original founders. Furthermore, we repeated the approaches after removing genetic profiles containing low-frequency alleles from a cluster, except if these alleles only occurred in the cluster under investigation.

## Results

**Fig 2:**
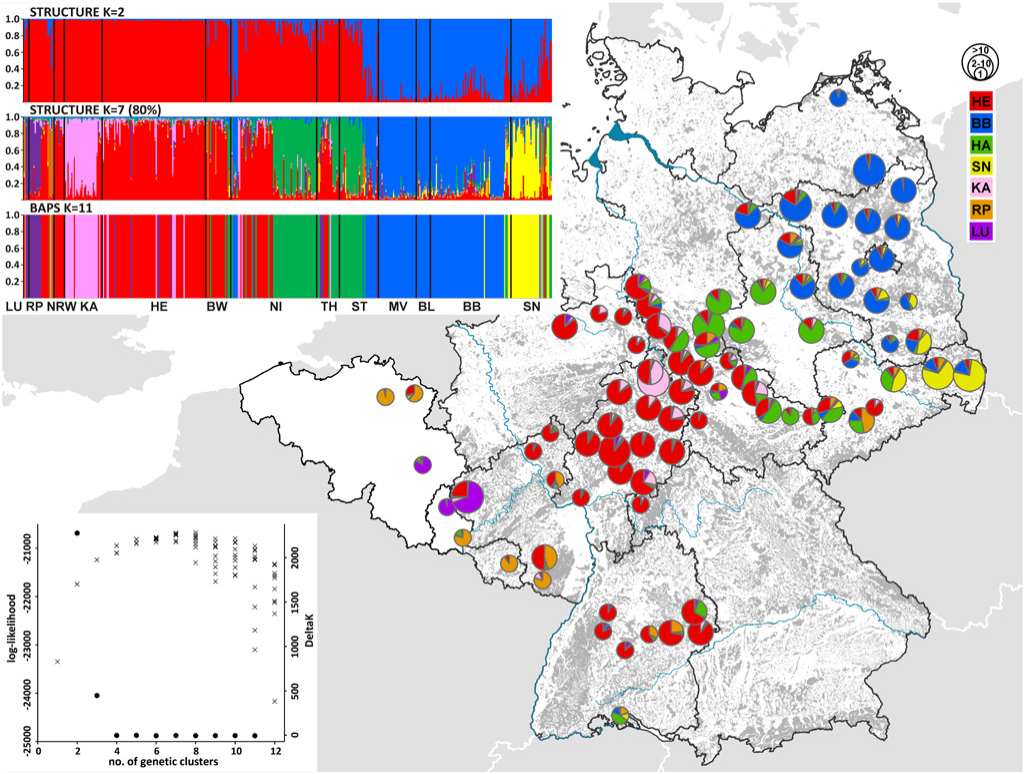
Geographic distribution of the STRUCTURE clusters (*K* = 7) for all 407 samples. The pre-defined populations correspond to the federal states of Germany. Pie charts represent the average per cluster assignment values for all the individuals in an administrative district and their size is indicative of the number of samples included. Light blue lines represent major rivers, grey pattern indicate forests. Top inset: Summary of the assignment analysis in STRUCTURE (*K* = 2, *K* = 7) and BAPS (K = 11). Each individual is represented by a single vertical line, representing the individual`s estimated proportion of membership to the genetic cluster. Colours correspond to the clusters in the main figure. The five BAPS-clusters of one to three individuals were coloured in different orange shades. Single samples from BY, SH and SL were included in the adjacent state. Bottom insert: Plot of the number of STRUCTURE clusters tested against their estimated log-likelihood (x) and DeltaK (•).

While *K* = 2 was identified as being the uppermost hierarchical level of structure when analyzing the STRUCTURE results with the *ΔK* statistic (Fig 2), log-likelihoods increased substantially beyond *K* = 2. While the highest values that converged well between runs were obtained for *K* = 6, the composition of the clusters differed between independent runs of *K* = 6 (S1 Fig). Eight of the ten independent runs of *K* = 7 converged on higher log-likelihood values and the clusters identified by these eight runs were consistent and compatible with the results obtained at *K* = 6 (S1 Fig). The structure represented roughly the following four main populations (1) Hesse and central Germany (HE), (2) the Harz Mountains and north-central Germany (HA), (3) Brandenburg and north-eastern Germany (BB) and (4) Saxony and eastern-central Germany (SN). Two of the remaining smaller clusters corresponded to individuals (5) from the city of Kassel in Hesse (KA) and from (6) an area including Luxembourg and its neighbouring regions (LU). The final cluster contained individuals from (7) northern Belgium and south-western and eastern Germany (RP). BAPS provided support for the presence of 11 genetic populations. However, five of these clusters were composed of single to three individuals only (all individuals from the STRUCTURE-RP cluster). The six remaining populations corresponded, in essence, to the first six STRUCTURE clusters introduced above (Fig 2). Subsequent results are based on the seven clusters inferred by STRUCTURE, using the eight converging runs of *K* = 7.

After excluding *PLO-M17* (see Materials and Methods), we did not find evidence for linkage disequilibrium between pairs of loci in the different clusters (*P*<0.0003). Furthermore, we did not observe any systematic deviations from Hardy-Weinberg expectations (HWE) at any of the remaining 20 loci. Between zero and five loci deviated from Hardy-Weinberg expectations (HWE) at *α* = 0.05-level in the seven clusters before multiple-test corrections. While no locus caused a problem in the HA, HE and SN clusters after correcting for multiple tests, three loci in the BB cluster (*P*<0.01; *PLOT-01*: *F*_IS_ = 0.30; *PLOT-04*: *F*_IS_ = 0.18; *PLM01*: *F*_IS_ = 0.31; remaining loci: -0.04<*F*_IS_<0.16) and one locus in both the LU (*P*<0.025; *PLO2-14*: *F*_IS_ = 0.50; remaining loci: -0.28<*F*_IS_<0.39) and RP (*P*<0.025; *PLOT-06*: *F*_IS_ = 0.58; remaining loci: -0.26<*F*_IS_<0.36) clusters exhibited a significant deficit of heterozygotes. In the urban KA cluster one locus exhibited a significant excess of heterozygotes (*P*<0.025; *PLM01*: *F*_IS_ = -0.29; remaining loci: -0.22<*F*_IS_<0.22)

**Fig 3:**
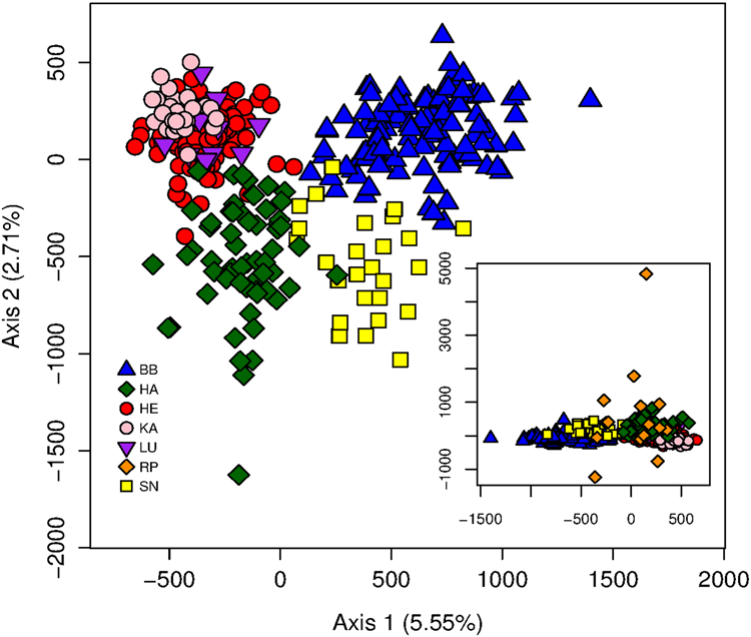
Factorial correspondence analysis of 393 raccoons assigned to the six main STRUCTURE clusters. Symbols and colors represent the different genetic clusters according to the STRUCTURE analysis. Inset: Factorial correspondence analysis with the seventh STRUCTURE cluster (RP) that mostly contained recently introduced individuals.

The FCA essentially separated BB and SN from all other populations along its first axis and SN and HA from all other populations along its second axis (Fig 3). While the four main populations (BB, HA, HE, SN) were clearly differentiated, populations LU and KA overlapped substantially with cluster HE. Individuals assigned by STRUCTURE to cluster RP were distributed all over the FCA graph and formed outliers in some cases (Fig 3).

In GENECLASS, seven of the 14 RP individuals were excluded with high probability from the other six clusters (*P* < 0.001) and another three individuals at the *P* < 0.01 level (the remaining four individuals: *P* ≤ 0.051). These results suggested that cluster RP (mostly) contained separately introduced individuals, thus all RP individuals were excluded from further analysis. No sample of the other six clusters could be excluded in a leave-one-out approach at the *P* < 0.001 level.

The six STRUCTURE clusters (excluding RP) were strongly differentiated, with *F*_ST_-values ranging between 0.049 and 0.166 (Table 1). The lowest estimates (*F*_ST_ ≤ 0.076) were obtained between HE and the three western-most clusters (HA, KA, LU) as well as between BB and SA. The AMOVA revealed that a significant part of genetic variation (10%; *P* < 0.001) was explained by the genetic clusters. According to the STRUCTURE results, all clusters had a relatively large degree of admixture (Fig 2). Only about half of all BB and HE individuals were assigned with *q* ≥ 0.9 to their respective clusters (BB: 43.9%; HE 48.0%). This proportion was even smaller in the remaining four clusters (LU: 15.4%; KA: 34.5%; SN: 19.2%; HA: 26.2%). The whole data set (excluding RP individuals) was characterized by a strong and significant IBD pattern (slope ± s.e. = -0.0245 ± 0.0034; *P*<0.001). However, when the analysis was limited to individuals within the same STRUCTURE cluster, the IBD pattern was still significant, but much weaker (slope ± s.e. = -0.0050 ± 0.0012; *P*<0.001).

**Table 1:** Pairwise *F*_ST_ values among STRUCTURE-defined genetic clusters.

	HE	BB	HA	SN	KA
BB	0.113				
HA	0.049	0.102			
SN	0.092	0.071	0.091		
KA	0.059	0.164	0.095	0.144	
LU	0.076	0.148	0.094	0.166	0.136

All values were significant (*P* < 0.001).

Estimates of allelic richness varied between 3.6 and 4.9, with the four main STRUCTURE clusters (BB, HA, HE, SN) all having estimates of *A*_R_ ≥ 4.3 (Table 2). The 13 individuals from cluster LU had the lowest heterozygosity values (*H*_O_ = 0.52; u*H*_E_ = 0.53). In all other clusters, *H*_O_ varied from 0.56 to 0.64 and u*H*_E_ from 0.58 to 0.65 (Table 2). BB and HE had a relative high number of private alleles (Table 2), with the most common private allele having a frequency of 0.101 and 0.017, respectively. The number of private alleles in clusters HA, LU and SN varied between one and five, with no private alleles occurring in the KA cluster.

**Table 2:** Comparison of genetic variability among STRUCTURE-defined genetic clusters.

Site	*N*	A	*A*_R_	*H*_O_	_u_ *H*_E_	_p_ *A*	freq_P_ *A*	*TrioML*	*N*_e_	95% CI
BB	114	6.8	4.9	0.56	0.60	9	0.004–0.101	0.116	305	210–523
HA	61	6.3	4.9	0.61	0.65	1	0.008	0.086	95	72–135
HE	150	7.0	4.3	0.61	0.62	13	0.003–0.017	0.093	413	249–1018
KA	29	4.1	3.7	0.60	0.58	0	-	0.223	902	85-infinite
LU	13	3.6	3.6	0.52	0.53	3	0.038–0.077	0.241	44	19-infinite
SN	26	5.4	4.7	0.64	0.63	5	0.019–0.173	0.138	168	70-infinite

*N* = number of samples, A = average number of alleles per locus, *A*_R_ = allelic richness (based on a minimal sample size of 13 diploid individuals), *H*_O_ & _u_ *H*_E_ = observed and unbiased expected heterozygosities, _p_ *A* = number of private alleles, freq_P_ *A* = frequency range of private alleles, *TrioML* = relatedness estimate, *N*_e_ = effective population size median calculated using NeEstimator, 95% CI: lower and upper 95% confidence intervals of *N*_e_ estimate.

The relatedness estimates TrioML calculated in COANCESTRY showed a high relatedness in the urban population KA as well as in the population at the range margin (LU) (Table 2). The effective population size estimates of KA, SN and LU had upper 95% confidence intervals that were infinite, suggesting that the corresponding point estimates were uninformative. Clusters HE and BB (which were the two known oldest introduction sites), had a higher effective population size than HA (Table 2).

As was to be expected, the total number of alleles included in the analysis affected the estimate of the minimum number of founders (Table 3). The estimates based on genotypes simulated by resampling from the allele frequency distribution (analysis 2) were inflated relative to the other estimates (Table 3). When resampling all alleles in the data sets (analysis 1), the inclusion of all individuals probably led to an overestimation of the number of the original founders of the two largest clusters BB and HE (97 or more). When removing all low-frequency alleles, we obtained estimates of 36 and 28 founders for BB and HE, respectively, while the corresponding estimates were 76 and 71 founders after removing non-private low-frequency alleles only. The estimates for the HA population varied between 28 and 53 founders, depending on the reference data set used. Also, the results of the allele-resampling approach suggest that the KA and SN populations may derive from fewer than 22 and 23 individuals, respectively. Finally, the genetic profiles simulated assuming on equal allele frequencies at all loci, always gave rise to the lowest number of founders, varying between 7 and 21 individuals in the LU and HE population, respectively.

**Table 3:** Estimates of minimum number of founders required to introduce all empirically observed microsatellite alleles into a population.

Cluster	Observed	Estimate minimum no. of founders based on		
	no. of alleles	resampling of alleles	allele freq.	equal freq.
(a)				
BB	135	97	213	18
HA	125	53	116	9
HE	140	133	309	21
KA	81	22	40	9
LU^1^	71	11	21	7
SN	108	23	52	13
(b)				
BB	107	36	48	-
HA	102	28	43	-
HE	82	28	35	-
KA^2^	72	13	20	-
SN^3^	81	10	21	-
(c)				
BB	125	76	150	-
HA	104	30	51	-
HE	102	71	154	-

Cluster = genetic clusters inferred using STRUCTURE. Estimates were obtained using three different approaches (see Materials and Methods): resampling of alleles (analysis 1), allele freq. (analysis 2), equal freq. (analysis 3). The analyses were performed using (a) the complete set of genotypes assigned to a cluster, (b) excluding genotypes containing low-frequency alleles (<0.02), (c) excluding genotypes containing non-private low-frequency alleles.

^1^no low-frequency alleles in population;

^2^no private alleles in population;

^3^all low-frequency alleles were private.

## Discussion

### Population genetic structure of raccoons in North America

The majority of the information on the large-scale genetic structure of North American raccoons is available for the Eastern United States [23]. When analyzing mtDNA diversity to evaluate the phylogenetic distinctiveness of four raccoon subspecies previously identified based on morphology, the authors identified 76 different control region haplotypes in a total of 311 samples [23]. The presence of three concordant lineages was inferred, which, however, did only partially correspond to the geographic ranges of the presumed sub-species. It was not possible to detect the geographic origin of German raccoons based on the analysis of mtDNA [22,23]. To the best of our knowledge, no comparable data has been published for microsatellite loci.

### Established populations and founder events

Our results contradict the common assumption that the whole German raccoon population descended from two separate introduction events in central and eastern Germany [14–16, 22]. The optimal partition solutions suggested by STRUCTURE and BAPS were very similar, as both algorithms provided evidence for the presence of six geographically coherent genetic populations in the study area, with further genetic units formed by recently introduced individuals (see below). The two largest and most widespread of these clusters were centered in Hesse and Brandenburg, suggesting that they stemmed from the known introductions in 1934 at the Edersee (HE) and 1945 in Wolfshagen (BB). Furthermore, we were able to confirm the existence of a genetic cluster in eastern Saxony (SN), which had already been suggested as another independent founder event based upon mtDNA data (Fig 1, [22]). Raccoons were, in essence, absent from Saxony until the mid-1990s [58] and Biedrzycka et al. [59] did not observe the same mtDNA haplotype (PLO16) in neighbouring Poland and the Czech Republic, suggesting a recent and independent origin of the SN population.

The fourth major genetic cluster was located in the Harz Mountains and in north-central Germany (HA). It has, indeed, been reported that up to 60 raccoons escaped from fur farms in 1945 in the Harz Mountains [18], however, it was not possible to confirm or reject its existence based on mtDNA data [22]. In the present study, the presence of a private allele in the HA population, the low overlap with other clusters in the FCA and a level of allelic richness comparable to the other three main populations support the hypothesis of an independent introduction. The relatively low genetic differentiation (in terms of *F*_ST_-values) between the HA and HE clusters probably resulted from recent admixture.

Both clustering methods also inferred the presence of two additional, smaller genetic clusters that were geographically coherent—the city of Kassel and Luxembourg/neighbouring regions—and therefore appeared to represent biologically meaningful units. The pattern of overlap in the FCA and the *F*_ST_-based genetic differentiation estimates suggested that these KA and LU clusters were founded by individuals from the HE cluster. Since the first raccoons were sighted in the city of Kassel in the 1960s [60], this urban population has reached the highest raccoon densities (> 100 animals per km^2^) in Europe [61]. A reduced genetic exchange between rural and urban populations has been shown for the Chicago metropolitan area [26] and the Kassel individuals did not carry any private alleles. It is therefore possible that raccoons in Kassel were genetically differentiated as a result of a founding event and/or limited exchange (leading to genetic drift) between urban and rural areas. In contrast to KA, the LU cluster had private alleles. In addition to natural dispersers from central Germany, raccoons from a distinct genetic population (e.g. recently released captive individuals) probably contributed the genetic make-up of the LU individuals.

However, there are alternative explanations for the inference of the clusters observed in our data set. For example, the pattern of IBD observed when considering the whole data set has the potential to cause the inference of artificial partitions by the clustering methods [62]. However, the within-clusters IBD pattern is relatively weak, suggesting the existence of genuine genetic discontinuities [63]. Furthermore, the best explanation for the observation that the different clustering methods reached similar conclusions, that all but one cluster had private alleles and that clusters BB, HA, HE, SN were clearly distinct in the FCA analysis, is that these four main clusters were biologically meaningful and indicative of four different founding populations. The two smaller clusters (LU, KA) overlapped in the FCA with the HE individuals, with KA also having no private alleles. Furthermore, the high relatedness coefficients observed for clusters KA and LU could also be a source of artificial clusters, as the inclusion of family members in a genetic data set can cause STRUCTURE to detect clusters that represent closely related family lineages [64]. We therefore cannot confidently exclude the possibility that the two smaller clusters were indeed artifacts of the clustering methods. Further sampling is required to clarify the genetic status of the raccoons in the city of Kassel and in the very west of our study area.

Finally, the results of the FCA and the GENECLASS exclusion tests provided substantial support for the hypothesis that the seventh STRUCTURE cluster (RP) essentially consisted of recently introduced individuals. These individuals formed five distinct clusters in the BAPS analysis, but were assigned to a single cluster by STRUCTURE. Even though three individuals excluded (at least) at the *P*<0.01-level, had one of the five mitochondrial haplotypes typical for Germany [22], our microsatellite results provide strong evidence for the ongoing release/escape of raccoons into the wild.

### Recent population admixture

We found strong evidence for recent admixture of the separate founder populations. It is perhaps surprising that some 70 years after the last major introduction event (not considering Saxony) the founder populations are still genetically distinct. While the inner-German border fortifications might have contributed to a reduced admixture between east and west, the core distribution areas of the eastern HA and BB clusters were relatively isolated until an increase in population density was detected since 1990 [65]. Given that the BB cluster still had the lowest overall rate of admixture, the river Elbe might have acted as a natural barrier, limiting raccoon dispersal [27]. Population admixture may have increased genetic diversity in each founding cluster and contributed to the significant increase in raccoon abundance over the last 20 years. Such an increase of genetic variation during biological invasions by combining genetic variation from multiple source populations has been described as a key factor in a number of successful invasions [2, 66].

### Genetic diversity

Even though not directly comparable to the present study due to differences in the suite of microsatellite used for genotyping, the average number of alleles observed in the two largest clusters (HE: *A* = 7.0; BB: *A* = 6.8) as well as the average heterozygosity values reported here (0.56 ≤ *H*_O_ ≤ 0.61 and 0.60 ≤ u*H*_E_ ≤ 0.62) were lower than comparable estimates from the raccoon’s native range [26–28]. The higher loss of diversity in the case of mitochondrial DNA [22] was probably caused by the different effective population sizes of both markers. The microsatellite diversity results are in line with our simulation analysis, which suggested that the number of founders was substantially larger than commonly assumed (see below). Despite the lower genetic diversity in the introduced compared to the native range, our study confirms that German raccoons have a relatively high genetic diversity compared to other invasive mammals. It is clear that the species is not an example of a genetic depauperate, but successful invader.

### Estimating the number of founders

While the number of individuals released can have a significant impact on the probability of invasion success [8] the presumed number of founders differed between the main clusters. According to historical records, HE might have been founded by as few as four individuals, BB by 25 and the HA population by 60 raccoons [13, 17, 18]. Our analyses partly deviate from these reported values. Some of our samples could even be excluded from the existing populations and must therefore be considered recently introduced individuals [42]. It is likely that, over the years, individuals kept as pets and escapees have entered the different populations [11].

The founder population size estimates based on resampling from the allele frequency distribution (analysis 2) were too high and very likely uninformative. In the subsequent discussion, we therefore focus the method based on re-sampling of the empirical data set (analysis 1). The highest estimates of founder individuals were obtained when all individuals (and their alleles) assigned to a cluster were considered in the analysis. Because of the recent admixture between the different founder populations, these estimates might well be adequate representations of the minimum number of individuals that have contributed to the overall genetic composition of the current clusters.

In order to attempt to infer the number of original founders, we removed all the individuals from a cluster whose genetic profiles contained alleles occurring at low frequency (< 0.02) [57]. The corresponding estimates suggested that the large BB cluster was founded by at least 36 individuals, while clusters HA and HE could have been founded by as few as 28 individuals. These results suggest that the two main populations HE and BB had a larger and HA a smaller number of founders than suggested by the historical record. The estimates for the number of founders would even be higher if one allows for the possibility that some of the low-frequency private alleles observed in BB and HE stemmed from the original founders but declined as a result of genetic drift. This latter scenario appears to apply to the younger SN cluster as all the low-frequency alleles were private. The analysis that included all the SN alleles therefore probably provided a more accurate estimate of founder population size (23 individuals) than the reduced data set.

The advantage of our new method is that it is very simple and does not require information on the allele frequencies in the source population(s). However, the estimates are affected by sample size and resampling from the allele frequency distribution clearly leads to overestimating founder numbers. Also the method cannot adequately account for genetic drift and does not allow for mutations creating new alleles. At the very least, the method provides some useful information regarding relative estimates of founder numbers (if sample sizes are broadly comparable). When we assumed all alleles in a cluster to have equal frequencies (to account for genetic drift), the BB estimate was in line with the historic information, while the HE cluster was estimated to have been founded by 21 individuals. In other words, it is likely that population HE at least had a larger number of founders than suggested by the historic record, even when considering the most conservative analysis.

Our study illustrates that it is precarious to fully trust the historical record of founder events in invasion biology. We proved the existence of at least four independent founding events with probably a substantially higher number of founders than commonly assumed.

## Supporting Information

S1 Fig Summary of the assignment analysis in STRUCTURE (*K* = 3 to *K* = 7). Each individual is represented by a single vertical line, representing its estimated proportion of membership to the different genetic clusters. Different independent STRUCTURE runs for the same value of *K* did not converge on the same solution (for *K* = 4 to *K* = 7). The percentage above each bar plot shows the proportion of the runs that converge on the presented clustering solution.
(TIF)

S1 Script R script used to estimate founder numbers based on re-sampling of alleles (analysis 1). (R)

S2 Script R script used to estimate founder numbers based on observed allele frequencies (analysis 2). (R)

S3 Script R script used to estimate founder numbers based on equal frequencies of the observed alleles (analysis 3). (R)
