# Supplementary figures and images for: Historical Invasion Records Can Be Misleading: Genetic Evidence for Multiple Introductions of Invasive Raccoons (Procyon lotor) in Germany
Source: PLoS One. 2015 May 6;10(5):e0125441. doi: 10.1371/journal.pone.0125441 (PMC4422738; doi:10.1371/journal.pone.0125441)

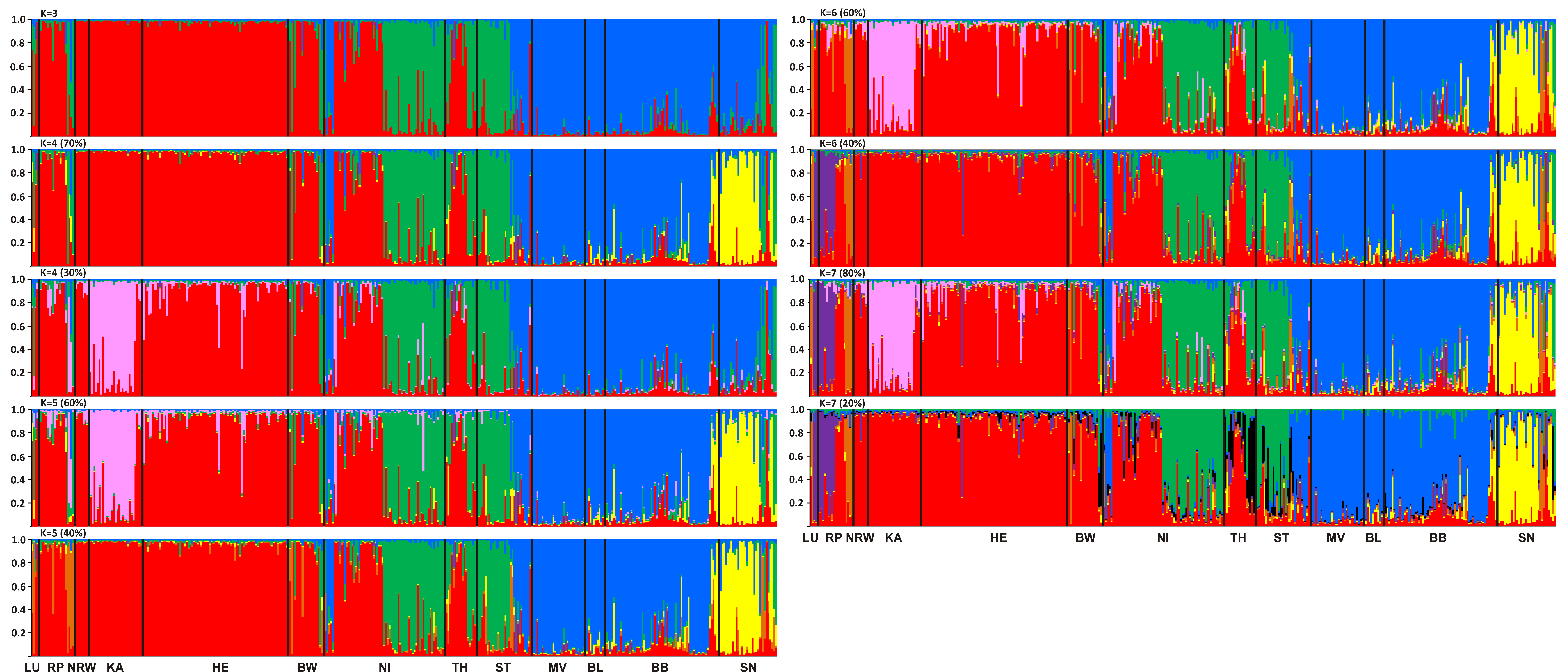

Supplement: S1 Fig — Each individual is represented by a single vertical line, representing its estimated proportion of membership to the different genetic clusters. Different independent STRUCTURE runs for the same value of K did not converge on the same solution (for K = 4 to K = 7). The percentage above each bar plot shows the proportion of the runs that converge on the presented clustering solution. (TIF) [file pone.0125441.s001.tif]
